# Digitoxin and its analogs as novel cancer therapeutics

**DOI:** 10.1186/2162-3619-1-4

**Published:** 2012-04-05

**Authors:** Hosam A Elbaz, Todd A Stueckle, William Tse, Yon Rojanasakul, Cerasela Zoica Dinu

**Affiliations:** 1 Department of Basic Pharmaceutical Sciences, West Virginia University, Morgantown, WV, USA; 2 Mary Babb Randolph Cancer Center, West Virginia University, Morgantown, WV, USA; 3 Department of Chemical Engineering, West Virginia University, Morgantown, WV, USA; 4 National Institute for Occupational Safety and Health, Morgantown, WV, USA; 5 Department of Basic Pharmaceutical Sciences, West Virginia University, PO Box 9530, 1 Medical Center Drive, Morgantown, WV 26506, USA; 6 Department of Chemical Engineering, West Virginia University, College of Engineering and Mineral Resources, PO Box 6102, ESB 445, Morgantown, WV 26506, USA

## Abstract

A growing body of evidence indicates that digitoxin cardiac glycoside is a promising anticancer agent when used at therapeutic concentrations. Digitoxin has a prolonged half-life and a well-established clinical profile. New scientific avenues have shown that manipulating the chemical structure of the saccharide moiety of digitoxin leads to synthetic analogs with increased cytotoxic activity. However, the anticancer mechanism of digitoxin or synthetic analogs is still subject to study while concerns about digitoxin's cardiotoxicity preclude its clinical application in cancer therapeutics. This review focuses on digitoxin and its analogs, and their cytotoxicity against cancer cells. Moreover, a new perspective on the pharmacological aspects of digitoxin and its analogs is provided to emphasize new research directions for developing potent chemotherapeutic drugs.

## Introduction

Cardiac glycosides (CGs) are a large family of chemical compounds found in several plants and animal species [1]. Plants containing CGs were used for more than 1500 years as diuretics, emetics, abortifacients, antineoplastics, and heart tonics [2]. In the 18th century, English physician and scientist William Withering discovered that a patient with congestive heart failure, "dropsy", improved after administering foxglove extract (*Digitalis purpurea L*.) [3]. Since then, many CGs have been isolated and their pharmacological effects have been tested; subsequently, CGs were used for treating congestive heart failure, cardiac arrhythmias, and atrial fibrillation [4,5].

**Figure 1:**
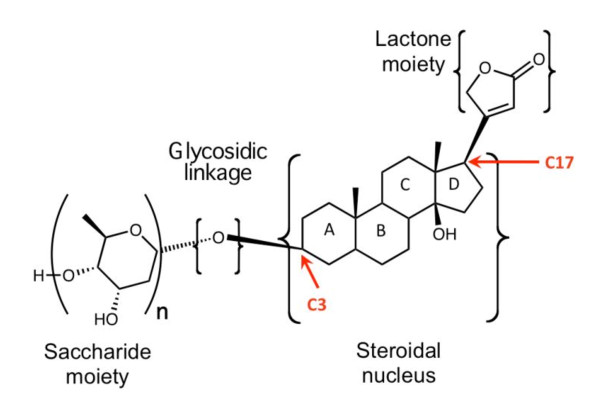
**Structural characteristics of CGs**. The common structural motif of a CG molecule is characterized by a steroidal nucleus, a lactone moiety at C17, and a saccharide moiety at C3 linked to the steroidal nucleus by a glycosidic linkage.

Generally, CGs share a common structural motif with a steroidal nucleus, a sugar moiety at position 3 (C3), and a lactone moiety at position 17 (C17) [1]. Figure 1 shows the common structural motif of CGs. The steroidal nucleus is the core structure of CG and is considered to be the active pharmacophoric moiety [6]. CGs also show an A/B and C/D cis-conformation that is different from mineralocorticoids, glycocorticoids, or sex hormones known to show trans-confirmation [6]. The presence of sugars at position 3 on the steroid ring significantly affects the pharmacological profile of each glycoside [7,8]. Free aglycones, for instance, show faster and less complex absorption and metabolism compared to their glycosylated counterparts [7]. Additionally, the type of sugar attached to the steroidal nucleus changes the potency of CG compounds [8,9].

It is well established that CGs inhibit Na^+^/K^+^-ATPase and increases intracellular sodium ions [10]. The Na^+^/K^+^-ATPase is a P-type pump that actively transports potassium ions inside and sodium ions outside cells in a 2:3 stoichiometry [11]. Such activity keeps intracellular sodium levels low, thus initiating and sustaining adequate electrochemical gradient across the plasma membrane [11]. Appropriate electrochemical gradient is essential for vital cellular processes such as ion homeostasis, neuronal communication, and apoptosis [10,12]. To maintain ion homeostasis after Na^+^/K^+^-ATPase inhibition by CGs, the cells have to restore intracellular sodium concentration to their basal levels by stimulating the Na^+^/Ca^2+ ^exchange pump to extrude sodium ions out of the cell and introduce calcium ions into the cell [10]. This activity increases intracellular calcium ions and results in increased cellular phenomena such as calcium dependent signaling and myocardial contractility [5].

## Cardiac glycosides and their anticancer action

A growing body of evidence suggests significant anticancer effects mediated by CGs. For instance, in the 8th century, CG plant extracts were used for treating malignant conditions [13]. An ancient Chinese remedy that employs an extract of *Bufo bufo *toad secretions contains several CGs and is still being used today for managing cancerous conditions [14-16]. Investigations in using CGs as anticancer drugs intensified in the 1960s [17,18]. Shiratori *et al. *examined the cytotoxicity of CGs in rodent cancer models and found inhibition of *in vitro *proliferation at concentrations that are relatively toxic to humans (0.1-10 μM) [17]. A series of landmark epidemiological studies by Stenkvist *et al. *compared breast cancer tissue samples from women maintained on digitalis (a CG) for cardiac conditions to tissue samples from control patients [19]. This study found that women on digitalis therapy developed more benign forms of breast tumors when compared to control patients [19-22]. Additionally, patients on digitalis treatment showed a 9.6-time lower cancer recurrence rate compared to control patients, 5 years after mastectomy for primary tumor [21]. In 1984, Goldin *et al. *examined 127 cancer patients treated with digitalis [23]. While patients in the control group had 21 cancer-related deaths, only one patient died in the digitalis group [23]. Twenty years later using follow-up data, Stenkvist *et al. *found that those patients on digitalis treatment showed significantly lower mortality rate than the control group [22]. Given the promising epidemiological results and the emergence of human cancer cell lines, several studies investigated the cytotoxic effect of different CGs on human cancer cells. Examples of the CGs of plant or animal origins that exhibit anticancer effects are shown in Table 1.

**Table 1:** Summary of most studied CGs and their anticancer activity

Compound	Structure and Natural Origin	Susceptible cancer types	IC_50 _Range	References
Bufalin	**	• Prostate cancer (PC3, DU145, and LNCaP cells) • Leukemia (THP1, U937, and MOLT-3 cells)	0.1-10 μM	[14,15,24-28]
Cinobufagin	**	• Prostate cancer (PC3, DU145, and LNCaP cells)	0.1-10 μM	[14,15,24,25,27,28]
Digitoxin	**	• Prostate cancer (PC3, DU145, and LNCaP cells) • Breast cancer (MCF-7 cells) • Renal adenocarcinoma (TK-10 cells) • Melanoma (UACC-62 cells) • Leukemia (K-562 cells) • Lung (A549 cells, and NCI-H460 cells)	0.01-10 μM	[9,29-40]
Digoxin	**	• Prostate cancer (PC3, DU145, and LNCaP cells) • Cervical cancer (Hela cells) • Lung (A549 cells, and NCI-H460 cells)	0.1-10 μM	[29-32,35,40,41]
Oleandrin	**	• Prostate cancer (PC3, DU145, LNCaP) • Leukemia (U-937, and HL-60 cells) • Breast cancer (MCF-7) • Lung • Malignant Fibroblast (VA-13) • Liver carcinoma (HepG2 cells) • Pancreatic cancer (PANC-1 cells)	0.1 - 1 μM	[42-57]
Ouabain	**	• Prostate cancer (PC3, DU145, LNCaP) • Breast cancer (MDA-MB-435scells) • Lung (NCI-H460 cells)	0.1-10 μM	[2,29,30,32-35,40,46,58-62]
Proscillaridin	**	• Breast cancer (MCF-7 cells) • Fibroblasts	30-100 nM	[29,30,32,33,35,43,63-66]

## Digitoxin: a well established CG

Digitoxin is one of the few CGs that have been extensively studied and have a well-established clinical profile [67,68]. Digitoxin is a cardiotonic drug with a narrow therapeutic window; thus, toxicity is a persistent concern whenever digitoxin is considered for therapy [69]. Cardiotoxicity is frequently encountered as the most significant toxicity following digitoxin's clinical administration [70,71]. However, digitoxin is perhaps one of the most promising anti-cancer CGs since it shows significant anticancer effect against several types of cancer including lung cancer, pancreatic cancer, leukemia, and breast cancer, all at therapeutic concentrations (*see *Table 1) [18,31,58,68,72]. Moreover, digitoxin has a prolonged half-life [69], which can potentially contribute to a less frequent and more stable pharmacokinetic profile in digitoxin-treated cancer patients. Such advantages qualify digitoxin for further laboratory investigations and clinical trials. However, to justify the anticancer clinical application of digitoxin, its anticancer mechanism ought to be clearly understood. Also, with the persistent concern about digitoxin's cardiotoxicity, further studies are needed to clarify the cytotoxic mechanism of digitoxin in cancer cells.

## Digitoxin and its anticancer mechanism

**Figure 2:**
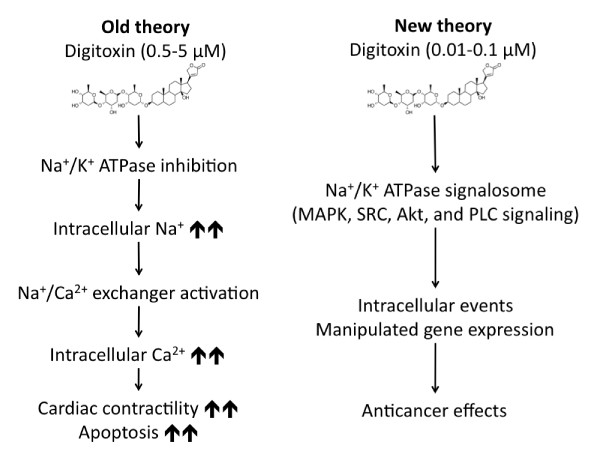
**Effects of digitoxin on Na^+^/K^+^-ATPase at micromolar and nanomolar concentrations**. (A) Old theory model summarizes the effect of digitoxin at 0.5-5 μM concentrations. (B) New theory model summarizes digitoxin's effect at 0.01-0.1 μM concentrations.

To understand its anticancer mechanisms, initial research focused on the ability of digitoxin to inhibit Na^+^/K^+^-ATPase pump at concentrations between 0.5 - 5 μM [5,10-12,73]. Such inhibition can result in increased intracellular calcium via increased Na^+^/Ca^2+ ^pump activity and subsequently induce apoptosis in cancer cells [74-76]. Figure 2A shows how inhibiting the Na^+^/K^+^-ATPase pump causes an accumulation of intracellular sodium, thus perturbation of cellular ion homeostasis [5,10-12]. For the cells to restore ion homeostasis, the Na^+^/Ca^2+ ^exchanger needs to be activated to export excess sodium to the extracellular space while importing calcium, which in turn causes an accumulation of intracellular calcium that mediates cellular events such as myocardial contractility, cytoskeleton remodeling, and apoptosis [5,10-12]. Interestingly, however, epidemiological studies have shown that digitoxin inhibits cancer cell viability at nanomolar concentrations (10-100 nM) [18,31,36,58]. This suggests that digitoxin exhibits a different mechanism for its anticancer effect than the one initially proposed.

In 2003, Xie *et al. *suggested that the signaling characteristics of Na^+^/K^+^-ATPase are distinct from the ion pumping activity [77]. It was shown that Na^+^/K^+^-ATPase signalosome is a multiple-protein signaling complex of 3 alpha (α) subunits and 2 beta (β) subunits that controls cellular activities like apoptosis [78], cell proliferation [79], cell motility [80], and tight junctions [81]. Subsequently, it was proposed that digitoxin at nanomolar concentrations activates Na^+^/K^+^ATPase signalosome to transmit intracellular signals. Figure 2B summarizes the intracellular effects of digitoxin upon binding to Na^+^/K^+^-ATPase at concentrations between 10-100 nM. Upon binding, digitoxin modulates the Na^+^/K^+^-ATPase protein complex activating the associated downstream signaling pathways, i.e., activation of several signaling cascades such as phospholipase C (PLC) signaling, mitogen-activated protein kinase (MAPK) signaling, phosphatidyl-inositol-3-kinase (PI3K) signaling, and Src kinase signaling.

The signaling cascades that are stimulated upon the interaction of digitoxin with Na^+^/K^+^-ATPase have mixed functions. For example, the MAPK signaling is mainly a proliferative and pro-survival signaling that can also be complemented by a pro-apoptotic signaling capability [82-88]. Src kinase signaling is a non-receptor tyrosine kinase that exhibits pro-survival as well as pro-apoptotic functions [89-97]. Therefore, digitoxin's ability to affect the downstream Na^+^/K^+^-ATPase signalosome is very complex. Such complexity, together with the digitoxin's narrow therapeutic window and its known cardiotoxicity, led to a slow progress in developing digitoxin as anticancer therapeutics [1,2,18,98].

## Designing digitoxin analogs as anticancer agents

**Figure 3:**
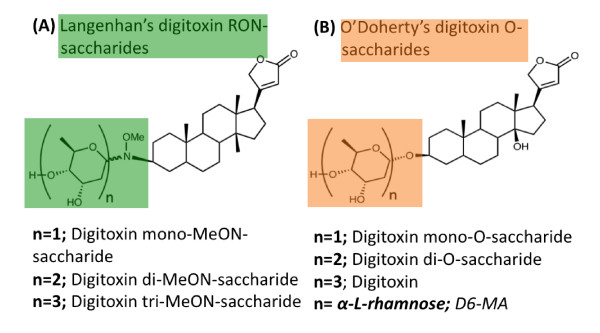
**The structure of novel digitoxin analogs**. (A) The common structural motif of a neoglycoside molecule developed by Langenhan *et al. *and characterized by a tertiary amine bond linking the saccharide moiety to the steroidal nucleus. (B) The common structural motif of a digitoxin O-saccharide molecule developed by O'Doherty *et al. *and characterized by an ether bond linking the saccharide moiety to the steroidal nucleus.

Early on, it was suggested that a potential approach to circumvent digitoxin's cardiotoxicity is to design synthetic analogs that are either more effective or less toxic than digitoxin when used as anticancer agents [8,99]. Such analogs were designed by structural modifications of digitoxin's chemical entity. For example, using digitoxin as a template, Langenhan *et al. *developed MeON-neoglycosylation, a chemoselective method for glycorandomization that employs modifying the glycosidic bond that links the saccharide moiety to the core steroidal nucleus of digitoxin [99]. In 2008, Zhou and O'Doherty developed another method for modifying the glycosidic linkage of digitoxin using palladium-catalyzed glycosylation [100,101]. The O'Doherty analogs are characterized by an ether linkage between the saccharide moiety and the steroidal nucleus. As a result, novel synthetic digitoxin O-glycoside analogs were synthesized [9,100,101]. Figure 3 shows the core structures of digitoxin analogs synthesized by Langenhan *et al. *and O'Doherty *et al. *respectively [99-101].

The differential potency and cytotoxic selectivity of digitoxin MeON-neoglycosides and digitoxin O-glycosides have been demonstrated in various cancer cell lines. For instance, Iyer *et al. *compared a library of digitoxin MeON-neoglycosides and digitoxin O-glycosides in a panel of 60 different cancer cell lines [9]. They showed that O-glycosides are more potent anticancer agents when compared to MeON-neoglycosides in a variety of cancer cell lines from leukemia, to lung, pancreatic and breast cancer cells. Additionally, the authors also show that the potency of the analogs depends on their sugar moiety with the monosaccharide analog being more potent than the di- and tri-saccharide analogs, respectively. Wang *et al. *used artificial O-monosaccharide analogs that they tested against a panel of 60 different cancer cell lines [37-39]. They identified three digitoxin monosaccharide analogs that showed significantly greater cytotoxic potential against non-small cell lung cancer (NSCLC) cells [37-39]. The most promising of these analogs, D6-MA, was subsequently evaluated for its anticancer mechanism by Elbaz *et al. *[36].

## Anticancer mechanism and selectivity of digitoxin and D6-MA analog

Elbaz *et al. *showed that the D6-MA analog was 4-5 folds more potent than digitoxin in inhibiting cell proliferation, inducing cell cycle arrest and apoptosis in NSCLC cells [36]. The authors also showed that digitoxin and D6-MA exhibited significantly greater cytotoxicity against NSCLC when compared to both primary and non-tumorigenic lung epithelial lines. The selective cytotoxicity in lung cancer cells included G2/M phase arrest and apoptosis [36]. Moreover, digitoxin and D6-MA inhibited the expression of p53, cdc2, cyclin B1, survivin, and Chk1/2, critical genes/proteins for cell proliferation.

**Figure 4:**
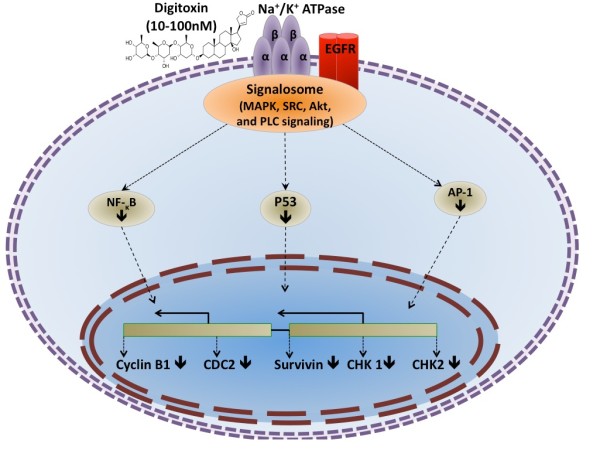
**Digitoxin modulates Na^+^/K^+^-ATPase signalosome and inhibits the transcriptional activity of AP-1 and NF-κB which mediate the expression of cancer-specific cycle regulatory genes such as cyclin B1, cdc2, Chk1, Chk2, and surviving**.

In order to explain the observed anticancer effect, it is postulated that digitoxin and D6-MA activate several transcriptional regulatory cascades via the Na^+^/K^+^-ATPase signalosome [61,62,77,102-107]. This suggested mechanism of selective cytotoxic effect may be associated with reduced expression of cell cycle regulatory proteins that are specifically overexpressed in cancer cells. Figure 4 illustrates how digitoxin regulates the expression of these cancer specific proteins.

The above suggested mechanism is supported by previous work showing that digitoxin inhibits AP-1 and NF-κB signaling through the Na^+^/K^+^-ATPase signalosome [45,55,106,108-112]. AP-1 is a transcription factor that promotes the expression of cell cycle regulatory proteins such as cdc2 and cyclinB1 [113-115]. Several studies have shown that cyclinB1 is essential for cell viability [116,117]. CyclinB1 and cdc2 are overexpressed in cancer cells [118-123] and form a complex with each other prior to mitosis. They catalyze chromatin condensation as well as nuclear envelope breakdown during mitosis [124,125]. The cyclinB1/cdc2 complex performs a rate limiting function in G2/M phase transition and protects mitotic cells from apoptosis by activating survivin. Survivin is a vital cell cycle regulatory protein known to be overexpressed in different cancer types [126-131]. Survivin controls cell progression through mitosis by promoting the chromosomal passenger complex and regulating microtubule dynamics [132-135]. Additionally, survivin regulates the mitotic spindle checkpoint [136-139]. Several studies suggest survivin downregulation as a biomarker for mitotic catastrophe [140-142]. Also, NF-κB transcription factor regulates the expression of cell cycle regulatory proteins such as survivin [143]. Thus, by inhibiting both AP-1 and NF-κB signaling, digitoxin and D6-MA potentially inhibit the expression of cdc2, cyclin B1, survivin and Chk1/2, as shown in Figures 2 and 4. Moreover, the association of cyclin B1/cdc2 complex with survivin indicates that the complex is crucial for G2/M phase transition and cell viability, and inhibiting its expression by both digitoxin and D6-MA could potentially explain the selective cytotoxicity of these compounds towards cancer cells. Since inhibiting Chk1/2 typically elicits checkpoint abrogation and uncontrolled cell cycle progression abolished cell viability [144-146], a potential alternative mechanism of digitoxin and D6-MA's cytotoxicity may be their ability to cause checkpoint abrogation. It is unclear, however, how digitoxin and D6-MA inhibit checkpoint kinase proteins in cancer cells. Distinguishing between CG-induced cell cycle arrest and/or checkpoint abrogation will improve our understanding on how CGs including digitoxin and its analogs exert their anti-proliferative/cell death effect.

## Conclusions and outlook

Digitoxin and D6-MA strongly modulate cell cycle machinery through the Na^+^/K^+^-ATPase signalosome in a manner that significantly undermines cell viability (Figure 4). However, several questions remain unanswered regarding their mechanistic control of cancer cell cycle. Delineating the mechanism of cell cycle arrest and selectivity of digitoxin and its analogs are particularly interesting. First, it will improve our understanding of how digitoxin and its analogs cause G2/M phase arrest in cancer cells. Second, it will help identify novel p53-independent therapeutic targets to mediate cancer cell death. Since many cancer types develop resistance to chemotherapy by reducing p53 expression and signaling [158-168], identifying p53-independent therapeutic targets will contribute to the development of new and more effective cancer treatments by circumventing p53 related resistance mechanisms. Third, detailing how digitoxin and D6-MA inhibit survivin expression in cancer cells will improve our understanding on how the drugs cause G2/M phase arrest and apoptosis. Last, dissecting the inhibitory effects of these compounds on survivin and p53 expression in cancer cells would explore whether and how the compounds induce mitotic catastrophe. Answering these questions will elevate our understanding on the antineoplastic mechanism of CGs at therapeutically relevant concentrations, and develop new and more effective cancer treatments [147].

## Disclaimer

Research findings and conclusions are those of the authors and do not necessarily represent the views of the National Institute for Occupational Safety and Health.

## Competing interests

The authors declare that they have no competing interests.

## Authors' contributions

HAE drafted the original manuscript; TAS and WT coordinated and helped to draft the manuscript. YR and CZD have been involved in revising the manuscript critically for its scientific content and have given final approval of the version to be published. All the authors read and approved the final manuscript.
